# Supplementary material for: Knowledge and attitudes towards stem cells and the significance of their medical application among healthcare sciences students of Jouf University
Source: PeerJ. 2021 Jan 19;9:e10661. doi: 10.7717/peerj.10661 (PMC7821763; doi:10.7717/peerj.10661)
Supplement: Supplemental Information 2 [file peerj-09-10661-s002.docx]

**Data Collection Proforma**

**Part 1: Demographic Details**

1. Age (in years):
2. Gender:
3. Male
4. Female
5. College (Specify):
6. Year of education
7. Smoking status:
8. Daily
9. Rarely
10. Non smoker
11. Marital Status:
12. Single
13. Married
14. Are you aware about Stem Cell Donor Registry
15. Yes
16. No

**2: Knowledge questions (Circle your response)**

| **S. No** | **Knowledge questions** | **Response** | |
| --- | --- | --- | --- |
| 1 | Stem cells are unspecialized. | True | False |
| 2 | Stem cells are capable of dividing and can self-renew for long periods. | True | False |
| 3 | Sperm and eggs are a source for adult stem cells. | True | False |
| 4 | Adult stem cells are also known as somatic stem cells. | True | False |
| 5 | Embryonic stem cells are capable of forming any cell type in the body including placenta. | True | False |
| 11 | Umbilical cord blood stem cells are embryonic stem cells. | True | False |
| 12 | Harvesting umbilical cord blood stem cells can cause pain and involve harmful risks to the newborn and mother. | True | False |
| 23 | Umbilical cord blood stem cell transplantation has a lower risk for graft versus host disease than other types of stem cells. | True | False |
| 14 | Autologous adult stem cell transplantation is controversial, primarily because of the immunogenic graft rejection. | True | False |
| 15 | Stem cells can be used to test new drugs and its effectiveness. | True | False |
| 16 | Bone marrow stem cells are taken from the spine. | True | False |
| 17 | Embryonic stem cell transplantation has a serious disadvantage as it could result in the formation of tumour. | True | False |
| 18 | Umbilical cord blood stem cell transplantation is less efficient compared  with bone marrow stem cell transplantation | True | False |
| 19 | Stem cells can be induced from normal skin cells by switching on genes controlling the pluripotent and differential of stem cells. | True | False |
| 20 | Stem cells are maintained by obligatory asymmetric replication | True | False |

**Part 3: Attitude questions (Circle your choice)**

| S. No | Attitude questions |  |  |  |  |  |
| --- | --- | --- | --- | --- | --- | --- |
| 1 | I am worried that stem cell transplantation might potentially open doors to human being killed for the benefit of others. | Strongly agree | Agree | Not sure | Disagree | Strongly disagree |
| 2 | The government should prohibit all researches regarding embryonic stem cells from embryo or aborted fetus. | Strongly agree | Agree | Not sure | Disagree | Strongly disagree |
| 3 | Life begins at conception; thus, embryonic stem cell research which involves the destruction of embryo is immoral, illegal and unnecessary. | Strongly agree | Agree | Not sure | Disagree | Strongly disagree |
| 4 | A blastocyst should be given the same respect and right to live as a living human adult. | Strongly agree | Agree | Not sure | Disagree | Strongly disagree |
| 5 | Stem cell transplantation should be widely practiced. | Strongly agree | Agree | Not sure | Disagree | Strongly disagree |
| 6 | I would advise pregnant mothers to store their umbilical cord blood stem cells for future purposes | Strongly agree | Agree | Not sure | Disagree | Strongly disagree |
| 7 | Competency in stem cell knowledge is important for me as a health care provider | Strongly agree | Agree | Not sure | Disagree | Strongly disagree |
| 8 | I am aware of the potential benefits, uses, and possible harms of stem cell research | Strongly agree | Agree | Not sure | Disagree | Strongly disagree |
| 9 | There should be more awareness program regarding stem cell. | Strongly agree | Agree | Not sure | Disagree | Strongly disagree |
| 10 | The future of mankind is bright if stem cell research could be successfully conducted. | Strongly agree | Agree | Not sure | Disagree | Strongly disagree |
